# Strabismus Recognition Using Eye-Tracking Data and Convolutional Neural Networks

**DOI:** 10.1155/2018/7692198

**Published:** 2018-04-26

**Authors:** Zenghai Chen, Hong Fu, Wai-Lun Lo, Zheru Chi

**Affiliations:** ^1^Department of Computer Science, Chu Hai College of Higher Education, 80 Castle Peak Road, Castle Peak Bay, Tuen Mun, NT, Hong Kong; ^2^Department of Electronic and Information Engineering, The Hong Kong Polytechnic University, Hung Hom, Kowloon, Hong Kong

## Abstract

Strabismus is one of the most common vision diseases that would cause amblyopia and even permanent vision loss. Timely diagnosis is crucial for well treating strabismus. In contrast to manual diagnosis, automatic recognition can significantly reduce labor cost and increase diagnosis efficiency. In this paper, we propose to recognize strabismus using eye-tracking data and convolutional neural networks. In particular, an eye tracker is first exploited to record a subject's eye movements. A gaze deviation (GaDe) image is then proposed to characterize the subject's eye-tracking data according to the accuracies of gaze points. The GaDe image is fed to a convolutional neural network (CNN) that has been trained on a large image database called ImageNet. The outputs of the full connection layers of the CNN are used as the GaDe image's features for strabismus recognition. A dataset containing eye-tracking data of both strabismic subjects and normal subjects is established for experiments. Experimental results demonstrate that the natural image features can be well transferred to represent eye-tracking data, and strabismus can be effectively recognized by our proposed method.

## 1. Introduction

Strabismus is a common ophthalmic disease that can lead to weak 3D perception, amblyopia (termed lazy eye as well), or even blindness if it is not timely diagnosed and well treated [1, 2]. More importantly, it has been shown that strabismus would cause serious psychosocial consequences in both children and adults [3–12]. These adverse consequences include education [5], employment [6], and dating [8]. Many young strabismic patients could be well treated if diagnosis and treatment were taken at their early ages. A preschool child's strabismus has a much larger chance to be cured than that of an adult. Timely diagnosis is thus essential. Traditional strabismus diagnosis methods, for example, cover test, Hirschberg test, and Maddox rod, are manually conducted by professional ophthalmologists. This would make the diagnosis expensive and drive people out of professional examinations consequently. Furthermore, ophthalmologists make decisions according to their experiences, and thus the diagnosis results are subjective. In view of that, we propose automatic recognition of strabismus in this paper. Automatic recognition of strabismus, which can be termed strabismus recognition as well, would perform strabismus diagnosis without ophthalmologists. As a result, the diagnosis results would be objective, and the diagnosis cost can be significantly reduced. We realize strabismus recognition by exploiting eye-tracking data, which are acquired using an eye tracker. The proposed eye-tracking-based strabismus recognition method allows us to build an objective, noninvasive, and automatic diagnosis system that could be used to carry out strabismus examination in large communities. For instance, we can place the system in a primary school such that the students can take their examinations at any time.

An eye-tracking technique has been successfully applied to solve various problems, for example, object recognition [13], content-based image retrieval [14], attention modeling [15], and image quality assessment [16]. But very little research on the eye-tracking technique for strabismus diagnosis has been reported. People have also proposed to leverage eye-tracking methodology for strabismus examination [17–20]. Pulido [17] employed the Tobii eye tracker to acquire gaze data to conduct ophthalmic examination including strabismus by calculating the deviation of gaze data. But Pulido proposed a method prototype only in [17]. The author had no real strabismic gaze data to demonstrate the prototype's performance. Model and Eizenman [18] proposed an eye-tracking-based approach for performing the Hirschberg test, a classical method to measure binocular ocular misalignment. But the performance of their approach was studied with five healthy infants only. The method's effectiveness for strabismus examination had not been tested. Bakker et al. [19] developed a gaze direction measurement instrument to estimate the strabismus angle. The instrument allows for unrestrained head movements. But only three subjects participated in the experiment. The number of subjects is relatively too small. Furthermore, there is no ground truth available for strabismic subjects. It is hence impossible to comprehensively evaluate the instrument's performance. In our previous work [20], we developed a system based on the eye-tracking technique to acquire gaze data for strabismus diagnosis. The diagnosis is performed by intuitively analyzing gaze deviations. But the system's effectiveness is verified by a strabismic subject and a normal subject only. In this paper, we developed a more effective eye-tracking system than that of [20] to acquire gaze data for strabismus classification. Instead of examining strabismus by directly analyzing gaze deviations in previous methods, we explore a machine learning method to realize strabismus classification. One big disadvantage of previous methods is that their accuracy is dramatically affected by every single gaze point. A noisy gaze point would cause an inaccurate examination result. By contrast, a learning method can eliminate the effect of a small number of noisy gaze points by using a large amount of data, so as to generate a more accurate result. Particularly, we leverage convolutional neural networks (CNNs), a powerful deep learning algorithm, to extract features from gaze data for strabismus recognition.

With the rapid developments of deep learning in recent years, the CNN has achieved numerous successes in computer vision and pattern recognition, for example, image classification [21], scene labeling [22], action recognition [23], and speech recognition [24]. With a hierarchical structure of multiple convolution-pooling layers, CNNs can encode abstract features from raw multimedia data. Especially for learning image features, CNNs have shown impressive performances. In our work, CNNs are exploited to generate useful features to characterize eye-tracking data for strabismus recognition. Concretely, a subject is asked to successively fixate on nine points. Meanwhile, the subject's eye movements are captured by an eye tracker. The eye-tracking data are then represented by a gaze deviation (GaDe) image which is produced according to the fixation accuracies of the subject's gaze points. After that, the GaDe image is fed to a CNN that has been trained on a large image database called ImageNet [25]. The output vectors of the full connection (FC) layers of a CNN are used as features for representing the GaDe image. Finally, the features are input to a support vector machine (SVM) for strabismus classification. It is expected that the image features of ImageNet learnt by CNNs would be well transferred to represent eye-tracking data for strabismus recognition. We build a gaze dataset using our eye-tracking system to demonstrate the proposed method's performance. The dataset is much larger than previously published strabismus datasets.

The rest of this paper is organized as follows.  Section 2 describes the methods exploited for strabismus recognition.  Section 3 introduces the dataset for experiments and reports experimental results.  Section 4 concludes this paper with final remarks. Before ending this introductory section, it is worth mentioning the contributions of this paper as follows:

- We develop an effective eye-tracking system to acquire gaze data for strabismus recognition.
- We propose a gaze deviation image to characterize eye-tracking data.
- We exploit convolutional neural networks to generate features for gaze deviation image representation.
- We demonstrate that natural image features learnt by convolutional neural networks can be well transferred to represent eye-tracking data, and strabismus can be effectively recognized by our method.

## 2. Methodology

### 2.1. The Proposed Strabismus Recognition Framework

**Figure 1:**
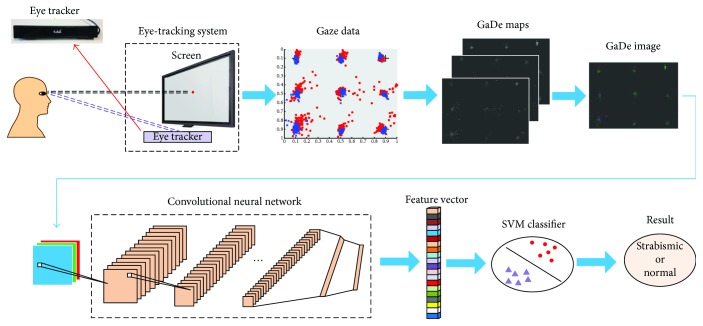
The proposed strabismus recognition framework.

Figure 1 shows our proposed framework for strabismus recognition. The recognition procedure is conducted as follows. First of all, the subject is asked to look at nine points respectively shown at different positions on a screen. Meanwhile, an eye tracker mounted below the screen detects the subject's eye movements and records his or her gaze points. The gaze data recorded by an eye tracker are then exploited to generate three gaze deviation (GaDe) maps, based on the fixation accuracies of left-eye gaze points, right-eye gaze points, and center points of two eyes, respectively. The three maps are combined to form a GaDe image with three maps denoting R, G, and B channels of the image. After that, the GaDe image is fed to a CNN which has been trained on ImageNet, so as to produce a feature vector for representing the GaDe image. Finally, the feature vector is fed to a SVM for classification, and the subject will be classified as strabismic or normal.

It is worth to present the motivations for the use of a CNN and GaDe image in our method before digging into the implementation details. We use the CNN to tackle our problems for two reasons. Firstly, eye-tracking gaze data are difficult to characterize. Up to now, there is still no standard feature for eye-tracking data representation. People have proposed some features such as fixation time and saccade path. But these features are designed for specific tasks. They are not suited for our strabismus recognition problem. Secondly, the CNN is powerful for learning discriminative features from raw images. It has shown state-of-the-art performance for various pattern recognition and image classification problems. We thus expect that the CNN can extract effective features for eye-tracking data representation. Since the CNN is good at extracting image features, we need to convert the raw gaze data to images before feature extraction by the CNN. That is why we propose GaDe images to represent the gaze data. The principle for us to design GaDe images is that the images should be able to well describe the difference between normal data and strabismic data. The details of eye-tracking data acquisition, GaDe image generation, and CNN models used will be presented in the following subsections.

### 2.2. Eye-Tracking Data Acquisition

We use the eye tracker Tobii X2-60 (shown in Figure 1) to acquire eye-tracking gaze data. Tobii X2-60 has a sampling rate of 60 Hz and tracking accuracy of 0.4 degree. Both of the sampling rate and tracking accuracy are high enough to precisely capture strabismic gaze data in our experiments. The eye tracker is adhered below the monitor of a laptop to build our eye-tracking system. The laptop is Lenovo ThinkPad T540p with a 1920 × 1080 screen resolution. The main reason for us to choose a laptop rather than a desktop for building the system is that it is convenient to carry the system in different environments for data acquisition. In order to position gaze points on the screen digitally, we need to define a coordinate system for the screen. The upper-left corner of the screen is set as the origin, the position value of which is (0,0), with horizontal line denoting *x*-coordinate and vertical line denoting *y*-coordinate. The values of the lower-right corner, upper-right corner, and lower-left corner are (1,1), (1,0), and (0,1), respectively. In other words, both *x* and *y* lie in interval (0,1) on the screen. We exploit Tobii Matlab SDK to develop our data acquisition interface.

Calibration needs to be performed before using the eye tracker to acquire gaze data. The purpose of calibration is to teach the eye tracking system the characteristics of the subject, such that the eye tracker can precisely detect the subject's eye movements. During the calibration, the subject is asked to fixate on a number of points displayed on the screen. In terms of the number of points used, we can have different calibration schemes, for example, one-point, three-point, or nine-point. We adopt a nine-point calibration scheme, as it can provide a high-tracking accuracy. The positions of the nine points on the screen are (0.1,0.1), (0.5,0.1), (0.9,0.1), (0.1,0.5), (0.5,0.5), (0.9,0.5), (0.1,0.9), (0.5,0.9), and (0.9,0.9). The result would be shown after each calibration. We can start real tracking tests if the calibration accuracy is acceptable. Otherwise, we should recalibrate.

**Figure 2:**
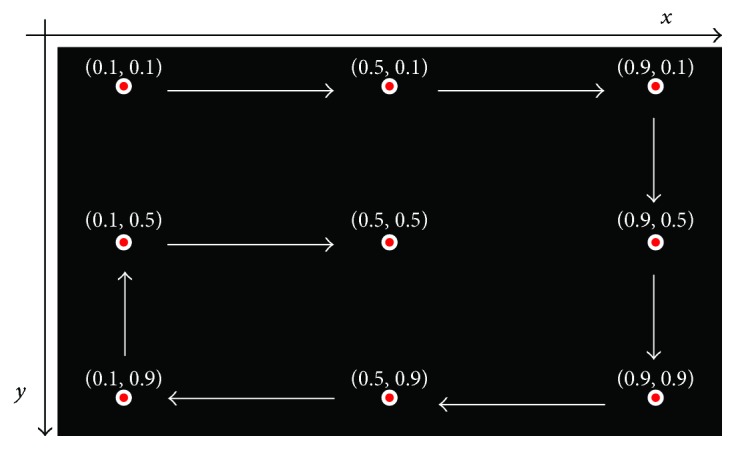
The nine-point gaze data acquisition interface.

A traditional method for ophthalmologists to examine strabismus is a nine-point method. The nine-point method is to ask the patient to fixate on nine target points at a certain distance in front sequentially. Meanwhile, the ophthalmologist observes the patient's eye movements. This method is able to comprehensively examine the patient's eye movements with rotations at different angles. Therefore, we adopt the same method to develop a gaze data acquisition interface. The nine points' positions are the same to the nine calibration points.  Figure 2 shows the nine-point interface. We use a black background, as it helps the subject to concentrate on the target points. A point is comprised by a red inner circle and a white outer circle. The radiuses of the inner circle and outer circle are 15 and 30 pixels, respectively. The points are displayed one by one orderly. The white arrows point out the display order. In a real test, the subject's position is adjusted to make sure that the subject is at a fixed distance (50 cm in our test) from the screen, and the subject's eye level and the screen center are in the same horizontal line. A distance of 50 cm is an optimal distance for the eye tracker Tobii X2-60 to track the subject's eye movements.

**Figure 3:**
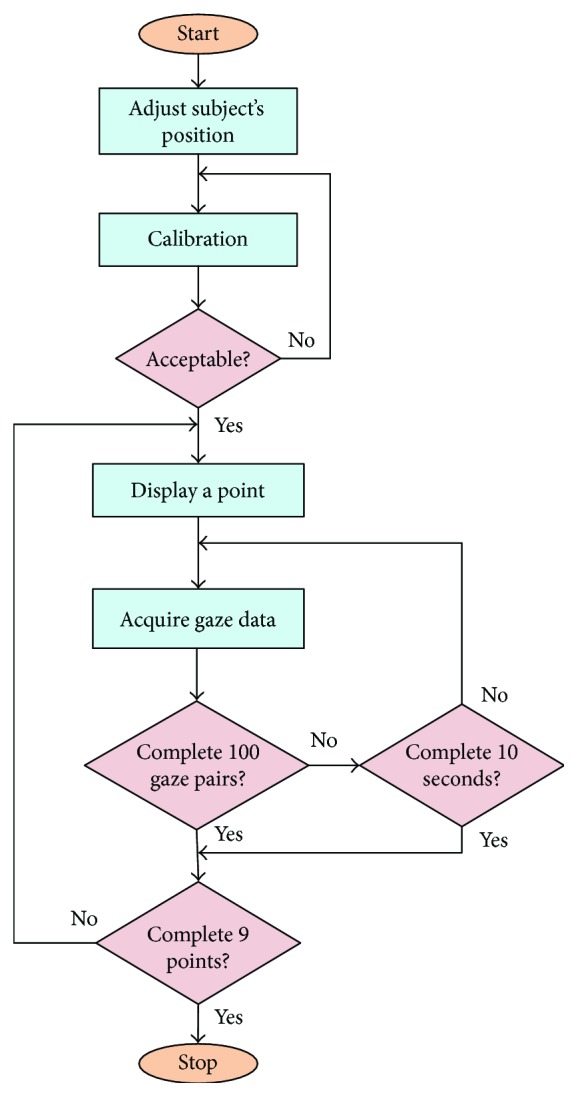
The gaze data acquisition procedure.

Figure 3 shows the procedure of gaze data acquisition. Each time one target point is displayed, the eye tracker records the subject's gaze points of both eyes simultaneously. The next target point would be displayed, if the number of effective gaze pairs acquired exceeds 100, where a gaze pair is defined as the two gaze points of two eyes captured by an eye tracker at one sampling moment and “effective” indicates that at least one gaze point of a gaze pair is located close enough to the target point. That is, the distance between the target point and either gaze point of a gaze pair must be smaller than a threshold (0.05 in this paper) predefined empirically. The distance is defined as Euclidean distance shown in Section 2.3. It is worth mentioning that for some serious strabismic subjects, it would be sometimes difficult to capture effective gaze points at some target points, in particular the points located at the four corners of the screen, because the strabismic subjects need to rotate the eyeballs to their extreme. In view of that, we let each target point display for at most 10 seconds for gaze data acquisition. The next target point would be displayed after 10 seconds no matter whether or not the system has collected 100 pairs of effective gaze points. Since the sampling rate of our eye tracker is 60 Hz, it would take only two seconds for collecting 100 gaze pairs from normal subjects. Hence, 10 seconds are long enough to capture gaze data for each point.

### 2.3. Gaze Deviation Image

The next step after gaze data acquisition is to generate a GaDe image to characterize the gaze data. To realize that, we need to first calculate three GaDe maps, which will serve as R, G, and B channels of the GaDe image, based on the fixation accuracies of two eyes' gaze points. Let *g*_*ij*_ denote the *i*th gaze pair for the *j*th target point and *p*_*ij*_^l^ = (*x*_*ij*_^l^, *y*_*ij*_^l^) and *p*_*ij*_^r^ = (*x*_*ij*_^r^, *y*_*ij*_^r^) denote the values of the left-eye gaze point and right-eye gaze point of gaze pair *g*_*ij*_, where 1 ≤ *j* ≤ 9 and 1 ≤ *i* ≤ 100, and superscripts l and r indicate left and right. Let *p*_*ij*_^t^ = (*x*_*j*_^t^, *y*_*j*_^t^) denote the *j*th target point's value. Then, we can have the fixation accuracies in terms of Euclidean distance for left-eye gaze point *p*_*ij*_^l^ as

(1) $$\begin{array}{c} d_{ij}^{l}=\sqrt{{\left(x_{j}^{t}-x_{ij}^{l}\right)}^{2}+{\left(y_{j}^{t}-y_{ij}^{l}\right)}^{2}}, \end{array}$$

and for right-eye gaze point *p*_*ij*_^r^ as

(2) $$\begin{array}{c} d_{ij}^{r}=\sqrt{{\left(x_{j}^{t}-x_{ij}^{r}\right)}^{2}+{\left(y_{j}^{t}-y_{ij}^{r}\right)}^{2}}. \end{array}$$

We defined the center of the gaze pair *p*_*ij*_^l^ and *p*_*ij*_^r^ as *p*_*ij*_^c^ = (*x*_*ij*_^c^, *y*_*ij*_^c^), which can be simply formulated as follows:

(3) $$\begin{array}{c} p_{ij}^{c}=\left(\frac{x_{ij}^{l}+x_{ij}^{r}}{2},\frac{y_{ij}^{l}+y_{ij}^{r}}{2}\right). \end{array}$$

Then, similar to (1) and (2), the fixation accuracy of the center *p*_*ij*_^c^ is calculated as

(4) $$\begin{array}{c} d_{ij}^{c}=\sqrt{{\left(x_{j}^{t}-x_{ij}^{c}\right)}^{2}+{\left(y_{j}^{t}-y_{ij}^{c}\right)}^{2}}. \end{array}$$

For one subject, we calculate the fixation accuracies *d*_*ij*_^l^, *d*_*ij*_^r^, and *d*_*ij*_^c^ for all of his or her gaze pairs. Based on the three types of fixation accuracies, three GaDe maps *M*^l^, *M*^r^, and *M*^c^ can be computed, respectively. The map size is equivalent to the input size of a CNN. In this paper, two map sizes (224^∗^224 and 227^∗^227) are adopted. The element values of the three maps are derived from three fixation accuracies *d*_*ij*_^l^, *d*_*ij*_^r^, and *d*_*ij*_^c^ of all the gaze pairs. One gaze pair represents one element in the three maps. In particular, the values of the gaze pair *g*_*ij*_ in the three GaDe maps *M*^l^, *M*^r^, and *M*^c^ are respectively calculated as

(5) $$\begin{array}{c} v_{ij}^{l}=round\left(\frac{d_{ij}^{l}}{max_{\left(i,j\right)}d_{ij}^{l}}\right)*255,\\ v_{ij}^{r}=round\left(\frac{d_{ij}^{r}}{max_{\left(i,j\right)}d_{ij}^{r}}\right)*255,\\ v_{ij}^{c}=round\left(\frac{d_{ij}^{c}}{max_{\left(i,j\right)}d_{ij}^{c}}\right)*255, \end{array}$$

where max(·) finds out the maximum value over all gaze pairs and round(·) rounds a value to its nearest integer. Equation (5) guarantees that the element values of the three GaDe maps are integers lying in interval (0,255), which is also the value interval of a real digital image. The positions of *v*_*ij*_^l^, *v*_*ij*_^r^, and *v*_*ij*_^c^ in maps *M*^l^, *M*^r^, and *M*^c^ are specified by (*x*_*ij*_^l^, *y*_*ij*_^l^), (*x*_*ij*_^r^, *y*_*ij*_^r^), and (*x*_*ij*_^c^, *y*_*ij*_^c^), respectively. The elements that are not associated with any gaze points in the three maps are assigned a value of 0. In the GaDe maps, big fixation deviations (far from the target point) possess big values. In other words, inaccurate gaze points play more important roles in a GaDe map. This makes sense, as the prominent difference between strabismic people and normal people is that strabismic people's one eye or even two eyes cannot well fixate on target objects. The three GaDe maps can thus effectively characterize the properties of strabismic gaze data. Generally, normal people's GaDe maps would have only a few bright (large intensity) points far from nine target points, and most relatively dark points are around the target points, while strabismic people's GaDe images usually have a large number of bright points located far from the target points. We combine three GaDe maps to form a GaDe image, with each map representing a color channel of the GaDe image. The GaDe image is then fed to a CNN for feature extraction.

### 2.4. Convolutional Neural Networks

A CNN is a hierarchical architecture that consists of a number of convolution layers and pooling layers. CNNs usually receive raw data, for example, image's pixels, as input and extract increasingly abstract features through hierarchical convolution-pooling layers. Take color image feature extraction as an example. An image's three-color channels are fed to the first convolution layer of the CNN. The convolution results, called convolution feature maps as well, are then downsampled in the pooling (e.g., max-pooling) layer following the first convolution layer, to generate pooling-feature maps. The pooling-feature maps are further passed to the next convolution layer and then to the pooling layer for processing. After a number of convolution and pooling operations, the feature maps are connected to an output layer through one or more FC layers. The FC layers can be used for classification like a multilayer perceptron, with the output vector representing different classes, or we can employ the outputs of FC layers as a feature vector of the input image and then use a classifier, for example, SVM, to perform classification on the feature vector. The hierarchical convolution and pooling operations make the features extracted by a CNN insensitive to transformation, rotation, and scaling.

We adopt six different CNN models that have been trained on ImageNet to generate features for representing eye-tracking gaze data. We use pretrained CNN models as feature extractors and do not train them using eye-tracking data in our work. There are two main reasons for us to do this. Firstly, we do not have enough eye-tracking data to well train a complicated CNN model. A CNN model may have thousands or even hundreds of thousands of weights that need to be trained. A large dataset is hence necessary to effectively tune so many weights. For instance, the CNN models we adopted have been trained on ImageNet, an image database that contains more than one million training images associated with 1000 classes. For strabismus classification problem, it is difficult to build a large dataset, since not so many strabismic people can be found to participate in the experiments. Actually, only 17 strabismic people participate in our experiments. It is, therefore, impractical to train a CNN model using such a few strabismic gaze data. However, it would be a good idea to employ a pretrained CNN as a feature extractor to generate features for gaze data representation. This will be demonstrated in Section 3. Secondly, the weights of CNN models are tuned using natural images rather than eye-tracking gaze data. We would like to investigate whether or not the information extracted from the natural image domain is applicable to the eye-tracking data domain. It would be meaningful if the features of natural images can be well transferred to represent eye-tracking data, since we would be able to make use of large quantities of natural images in the internet to help generate features, rather than to manually design complicated algorithms to extract features, for eye-tracking data representation. The six CNN models we adopted are named AlexNet [21], VGG-F, VGG-M, VGG-S [26], VGG-16, and VGG-19 [27]. All of them have three FC layers but different numbers of convolution layers. Their differences also lie in input size, number of convolution filters in each layer, max-pooling size, and so on. People can refer to [21, 26, 27] for the architecture details of the six models. The six models have the same three FC layers. The first two FC layers use ReLU [28] transfer function, and the final FC layer adopts Softmax transfer function. For each FC layer, we employ the input vector and output vector of the transfer function as feature vectors of GaDe images. Then, we can extract in total six feature vectors from three FC layers. The six feature vectors are denoted by **l**_1_, **l**_2_, **l**_3_, **l**_4_, **l**_5_, and **l**_6_, and their dimension sizes are 4096, 4096, 4096, 4096, 1000, and 1000, respectively. We will compare the performances of six feature vectors for six CNN models in Section 3. Note that the input size of AlexNet is 227 × 227, while the input sizes of the other five models are all 224 × 224. Therefore, the GaDe images need to be resized to 227 × 227 for AlexNet and 224 × 224 for the other five models.

### 2.5. Baseline Method

In order to demonstrate the effectiveness of CNNs for extracting features from gaze data for strabismus recognition, we propose a baseline method for a comparison. The baseline method models the normal gaze points of each target point as a multivariate Gaussian distribution. The parameters (mean vector and covariance matrix) of each Gaussian distribution are calculated using the normal training data. To construct the Gaussian distribution, we represent a gaze pair *g*_*ij*_ by the *x*-coordinate differences and *y*-coordinate differences between the target point *p*_*j*_^t^ and the pair's two gaze points *p*_*ij*_^l^ and *p*_*ij*_^r^ as follows:

(6) $$\begin{array}{c} u_{ij}={\left[x_{j}^{t}-x_{ij}^{l},y_{j}^{t}-y_{ij}^{l},x_{j}^{t}-x_{ij}^{r},y_{j}^{t}-y_{ij}^{r}\right]}^{T}. \end{array}$$

Then, we can have the Gaussian probability density function for the gaze pair *g*_*ij*_ as

(7) $$\begin{array}{c} p\left(u_{ij};\mu_{j},\underset{j}{\Sigma }\right)=\frac{1}{{\left(2\pi \right)}^{2}\sqrt{\left|\Sigma_{j}\right|}}\exp\left(-\frac{1}{2}\left(u_{ij}-\mu_{j}\right)\overset{-1}{\underset{j}{\Sigma }}\left(u_{ij}-\mu_{j}\right)\right), \end{array}$$

where **μ**_*j*_ and Σ_*j*_ are the mean vector and covariance matrix of the Gaussian distribution for the *j*th target point, respectively. **μ**_*j*_ and Σ_*j*_ are calculated using the normal training gaze pairs that belong to the *j*th target point. |Σ_*j*_| computes the determinant of Σ_*j*_.

The baseline method performs classification as follows. Given the gaze pair *g*_*ij*_, if its density value in (7) is larger than the threshold *α*_*j*_, the gaze pair is classified as normal. If the proportion of normal gaze pairs is larger than the threshold *β*_*j*_, then the target point is classified as normal for the subject. Otherwise, the target point is classified as strabismic for the subject. If one of the right target points is classified as strabismic, the subject will be finally classified as strabismic. In other words, a normal subject should possess normal fixations on all nine different directions. Once the fixation on one direction is abnormal, the subject will be diagnosed as strabismic. This is reasonable, since some types of strabismus such as incomitant strabismus may fixate poorly on a specific direction only. In medical examination, a subject may be also diagnosed to have strabismus once the ophthalmologist observes that the subject's two eyes do not align at a specific direction. Thresholds *α*_*j*_ and *β*_*j*_ are learnt using grid search, such that the classification accuracy on the training data is maximized.

## 3. Experiments

### 3.1. Eye-Tracking Gaze Data

We cooperated with Hong Kong Association of Squint and Double Vision Sufferers to collect strabismic data. In total, 17 members of the association suffering from strabismus consented to participate in our experiments. In addition to the 17 strabismic subjects, we invited 25 normal subjects to join in our study. All subjects are adults, with age ranging from 25 to 63, including both male and female. They have been diagnosed by a professional ophthalmologist, and the diagnosis results are used as ground truth in this paper. After ethics approval and informed consent, the 42 subjects followed the data acquisition procedure introduced in Section 2.2 to participate in our experiments, and finally, we collected 42 eye-tracking samples. The 17 strabismic subjects suffer from different types of strabismus (e.g., recessive, intermittent, and manifest) in various severities (e.g., mild, moderate, and severe). Recessive strabismus is only present when binocular vision has been interrupted, such as covering one eye. This type of patients can still maintain fusion. The patients are usually aware of having recessive strabismus after taking examination by an ophthalmologist. Manifest strabismus can be observed while a patient looks at an object binocularly. Intermittent strabismus is a combination of recessive strabismus and manifest strabismus. People suffering from recessive strabismus, intermittent strabismus, and mild manifest strabismus are sometimes difficult to be distinguished from normal people apparently, as their fixation deviations are small, especially for recessive strabismus and intermittent strabismus.

**Figure 4:**
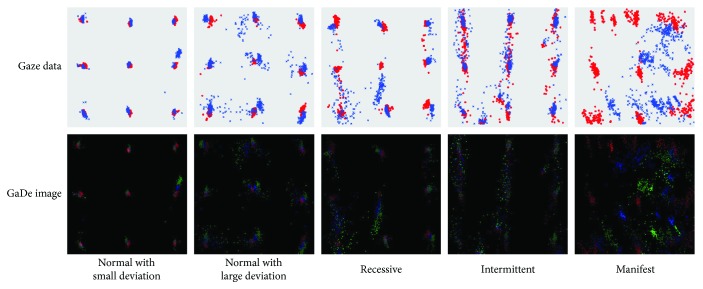
Examples of gaze data and corresponding GaDe images, where the first two columns represent normal data with small deviation and large deviation and the third, fourth, and fifth columns represent data of recessive strabismus, intermittent strabismus, and manifest strabismus, respectively.

Figure 4 shows some examples of gaze data and GaDe images. The first row displays all gaze points for nine target points in a map, with red ∗ denoting left gaze points and blue × denoting right gaze points. The second row shows the corresponding GaDe images for the gaze data of the first row. For a better visualization, the GaDe images have been brightened by adding a number (50) to the gaze points' values in the images. The first two columns are two normal samples, one with good fixation (small deviation) and one with relatively poor fixation (large deviation). The other three columns from left to right represent strabismic samples of recessive strabismus, intermittent strabismus, and manifest strabismus, respectively. Note that the colors in the first row represent the left and right gaze points, and the colors in the second row represent the R, G, and B channels of GaDe images. The two main observations can be drawn from Figure 4. Firstly, gaze points with large deviations shown in the first row are highlighted in the corresponding GaDe images, and those with small deviations are inhibited. Therefore, inaccurate gaze points would contribute more in recognizing strabismus using GaDe images. Secondly, the data distributions of normal sample with small deviation and manifest sample are distinctive. They are easy to distinguish. By contrast, the data distributions of normal sample with large deviation and recessive or intermittent sample look similar. It is difficult to distinguish them intuitively. That is why we exploit CNNs to solve the problem. We expect that CNNs as a powerful abstract feature extractor can extract distinctive features from different samples, so as to effectively classify normal data and strabismic data. It is worth mentioning that we focus on binary strabismus classification rather than recognizing different types of strabismus in this paper. The major reason is that we do not have enough data for each strabismus type at this moment. However, we consider that CNNs could extract useful features from GaDe images of different strabismus types in case that sufficient data are provided, and then the proposed method would be well applied to recognizing strabismus types. We leave this task for future work when we acquire sufficient data for different strabismus types.

### 3.2. Experimental Results

We have in total 42 samples, with 25 normal samples and 17 strabismic samples. A leave-one-out evaluation scheme is adopted. That is, each time one sample is used for testing, and the rest 41 samples are for training. We can thus have 42 different results. The 42 results are averaged to get the final performance. LIBSVM [29] is employed to implement SVM classification. A linear kernel of a SVM is used for both the CNN method and baseline method, and the SVM classifiers are trained using a two-fold cross validation scheme.  Table 1 tabulates the classification accuracies of six CNN models (by row) using different feature vectors (by column) extracted from three FC layers. The final column represents the concatenation of all the six feature vectors as a one feature vector. The accuracy of the baseline method is 69.1. As can be seen from Table 1, the features extracted from VGG-S overall perform the best. The highest accuracy (95.2%) is achieved when the feature vector **l**_3_ of VGG-S is used. Feature vectors **l**_1_, **l**_2_, **l**_3_, and **l**_4_ outperform feature vectors **l**_5_ and **l**_6_ for most of the cases. One possible reason is that **l**_1_, **l**_2_, **l**_3_, and **l**_4_ extracted from the first two FC layers contain richer features than **l**_5_ and **l**_6_. Note that the concatenation of all feature vectors sometimes obtains lower accuracy than that of some individual feature vectors, as shown in the final column. The most important finding from Table 1 is that the features extracted from six CNN models perform much better than the baseline method, except for some cases when the feature vector **l**_6_ is used. This indicates that the CNN features can effectively characterize the GaDe images derived from eye-tracking data. CNN features can be a promising representation of eye-tracking data.

**Figure 5:**
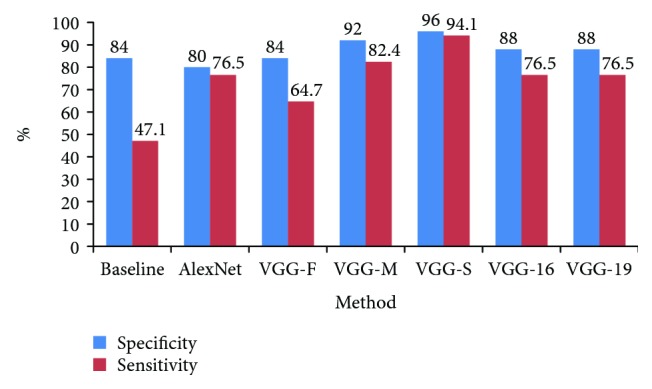
Specificity and sensitivity of different methods.

Specificity and sensitivity are two important metrics to measure the performance of a medical classification method. A good method should have high values for both specificity and sensitivity. For our strabismus recognition problem, specificity is defined as the percentage of normal subjects who are correctly classified as normal and sensitivity is defined as the percentage of strabismic subjects who are correctly classified as strabismic. In order to study the specificity and sensitivity of our method, for each CNN model, we select the result with the highest classification accuracy. According to Table 1, the highest accuracies for AlexNet, VGG-F, VGG-M, VGG-S, VGG-16, and VGG-19 are 78.6 (**l**_1_), 81.0 (column “All”), 88.1 (**l**_1_), 95.2 (**l**_4_), 83.3 (**l**_1_), and 83.3 (column “All”), respectively. We show the specificity and sensitivity of the six CNN results as well as the baseline method in Figure 5. Evidently, VGG-S possesses the best specificity and sensitivity. Only one normal subject and one strabismic subject are misclassified by VGG-S. The baseline method has a high specificity (84%) but a very low sensitivity (47.1%). This means that the baseline method is insensitive to strabismic data. It tends to classify the data as normal. By contrast, the difference between specificity and sensitivity of CNN features is relatively small, especially for VGG-S. This substantiates two things. Firstly, the proposed GaDe images are able to effectively characterize both normal gaze data and strabismic gaze data. The two types of eye-tracking data can be well separated by GaDe images. Secondly, the natural image features learnt by CNNs can be well transferred to represent GaDe images.

Overall, the experimental results have demonstrated that the proposed method is a promising alternative for strabismus recognition. In the future, the accuracy can be improved in two major ways. One way is to employ more advanced pretrained CNN models for better feature extraction. The other way is to collect more gaze data, especially data of different strabismus types. With sufficient data, we would then be able to fine-tune CNN models, as a result of which CNN models could learn more discriminative features to boost the classification accuracy.

## 4. Conclusion

In this paper, we first design an eye-tracking system to acquire gaze data from both normal and strabismic people and then propose a GaDe image based on gaze points' fixation deviation to characterize eye-tracking data. Finally, we exploit CNNs that have been trained on a large real image database to extract features from GaDe images for strabismus recognition. Experimental results show that GaDe images are effective for characterizing strabismic gaze data, and CNNs can be a powerful alternative in feature extraction of eye-tracking data. The effectiveness of our proposed method for strabismus recognition has been demonstrated.

## Figures and Tables

**Table 1:** Accuracies (%) of different CNN models. The accuracy of the baseline method is 69.1.

Feature	**l**_1_	**l**_2_	**l**_3_	**l**_4_	**l**_5_	**l**_6_	All
AlexNet	78.6	78.6	76.2	76.2	73.8	76.7	76.2
VGG-F	76.2	76.2	76.2	76.2	78.6	65.1	81.0
VGG-M	88.1	85.7	85.7	85.7	78.6	57.1	78.6
VGG-S	85.7	81.0	78.6	**95.2**	76.2	79.1	83.3
VGG-16	83.3	81.0	76.2	81.0	76.2	67.4	83.3
VGG-19	81.0	78.6	81.0	81.0	71.4	62.8	83.3
